# Precursor-Driven Catalytic Performances of Al_2_O_3_-Supported Earth-Abundant Ni Catalysts in the Hydrogenation of Levulinic Acid and Hydroxymethylfurfural into Added-Value Chemicals

**DOI:** 10.3390/molecules29132963

**Published:** 2024-06-21

**Authors:** Marcin Jędrzejczyk, Emilia Żyłka, Karolina Chałupka-Śpiewak, Agnieszka M. Ruppert

**Affiliations:** Institute of General and Ecological Chemistry, Lodz University of Technology, Żeromskiego 116, 90-924 Łódź, Poland; emilia.zylka1991@gmail.com (E.Ż.); karolina.chalupka@p.lodz.pl (K.C.-Ś.)

**Keywords:** nickel catalyst, nickel precursor, levulinic acid, hydroxymethylfurfural, hydrogenation, metal support interaction

## Abstract

It has been shown that the nature of the metal precursor and the thermal effects during calcination determine the physicochemical properties of the catalysts and their catalytic activity in the levulinic acid (LA) and 5-hydroxymethylfurfural (HMF) hydrogenation reactions. The endothermic effect during calcination of the inorganic nickel precursor promoted higher metal dispersion and stronger interaction with the alumina surface. In contrast, the exothermic effects during the calcination of organic nickel precursors resulted in smaller metal dispersion and lower interaction with the support surface. A clear relationship was found between the size of the metal crystallites and the yield of LA hydrogenation reaction. The smaller crystallites were more active in the LA hydrogenation reaction. In turn, the size of the metal particles and their nature of interaction with the surface of the alumina influence the hydrogenation pathways of the HMF.

## 1. Introduction

**Scheme 1:**
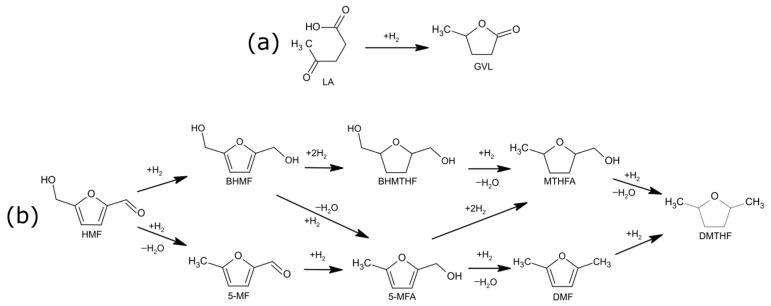
Reaction pathways of (**a**) LA hydrogenation and (**b**) HMF hydrodeoxygenation.

Nowadays, due to the growing energy demand, limited fossil fuel stocks and increasing environmental awareness, new economically viable routes to sustainable sources of chemicals and energy are investigated [1]. Biomass is a source of bio-derived compounds called “platform molecules” that can be applied in various branches of the chemical industry [2]. Levulinic acid (LA) and 5-hydroxymethylfurfural (HMF) are among the most valuable compounds due to their use in the synthesis of chemicals, biofuels and polymers from renewable resources [3,4]. The reaction pathways of LA hydrogenation and HMF hydrodeoxygenation are shown in the Scheme 1. γ-Valerolactone (GVL) is formed as a result of LA hydrogenation via the reduction of the carbonyl group and acid-catalyzed cyclization [5].

On the other hand, the hydrodeoxygenation of HMF gives 5-methylfurfural (5-MF) or 2,5-bis-hydroxymethylfuran (BHMF), respectively. The hydrogenation of the furan ring of BHMF leads to 2,5-bis-hydroxymethyltetrahydrofuran (BHMTHF). 5-Methylfurfuryl alcohol (5-MFA) is formed as a result of the dehydration of BHMF or the hydrogenation of 5-MF. 5-MFA is then either hydrogenated to 5-methyltetrahydrofurfuryl alcohol (MTHFA) or dehydrated to DMF (2,5-dimethylfuran). The hydrogenation of DMF leads to 2,5-dimethyltetrahydrofuran (DMTHF) that can also be obtained via the hydrogenolysis of BHMTHF or MTHFA [6,7].

The γ-valerolactone can be used as a raw material in the production of liquid fuels, monomer in the synthesis of polymers, intermediate or solvent in the syntheses of fine chemicals [8]. The HMF hydrogenation products are also used in the synthesis of polymers (2,5-bishydroxymethyltetrahydrofuran), as a fuel additive (2,5-dimethyl furan) or solvent (2,5-dimethyltetrahydrofuran) [9].

Catalysts based on non-noble metals are sought as an excellent alternative to noble metal systems [10,11] due to their long-term availability, lower costs and environmental friendliness. Ni-based materials are efficient hydrogenation catalysts applied to LA and HMF, and their properties strongly depend on different factors. From one hand, the metal dispersion is a deciding factor for reaching high activity performance. Jiang et al. shown that Ni/MgO-Al_2_O_3_ catalysts are very active in LA hydrogenation thanks to the high metal dispersion reached on the bi-oxide support in comparison to both MgO and Al_2_O_3_ counterparts [12]. Furthermore, in HMF hydrodeoxygenation, the selectivity of the Ni catalysts could be tuned depending on the Ni dispersion directing the pathway through BHMF (larger crystallites) or 5-MF (smaller ones) [13]. Therefore, the presence of finely dispersed Ni nanoparticles on a mesoporous carbon-rich material showed an efficient catalytic ability for the hydrogenolysis of HMF to DMF with very high selectivity.

On the other hand, very high activity in LA hydrogenation was explained by the coexistence of metallic nickel together with acid sites in the case of montmorillonite-supported Ni catalysts [14]. Lewis acid sites may facilitate the formation of GVL via the angelica lactone intermediate, while on the other side the Bronsted acid sites can initiate the ring opening reaction and the formation of further hydrogenation products like valeric acid [15]. Similarly, this factor was also considered of prime importance in HMF dehydrogenation. Kong et al. demonstrated the relationship between both metal and acid sites and the product distributions in the HMF hydrodeoxygenation. The presence of balanced Ni and acid sites facilitated the DMF production, whereas an excessive presence of metal sites promoted C=C hydrogenation, leading to over-hydrogenation products [13,16]. Furthermore, the presence of too strong acid sites could stimulate the parallel adsorption of the furan ring to induce ring saturation whereas weak acid sites could facilitate a tilted adsorption configuration, and in consequence ring opening [17]. Furthermore, the Ni availability and the metal–support interaction were often considered as key factors tuning the catalyst performance, and too strong interaction of Ni with alumina and formation of possible spinel-like structures were reported as being negative in both hydrogenation reactions [18,19]. On the other hand, the metal–support interaction in the case of Ni/Al_2_O_3_ was tuned by the addition of another oxide, for example CeO_2_, which limits its strength. This had a positive impact on the reaction performance and enabled achieving higher yields to BHMTHF [20].

Thus, the activity of Ni catalysts in LA and HMF hydrogenation reactions can be strongly modified by its physiochemical properties. The most important seem to be nickel dispersion and its particle size, metal–support interaction, and the amount of acid centers on the catalyst surface. It is therefore of high importance to understand to what extent it is possible to tune and control the catalyst properties. The choice of the Ni precursor at the preparation stage enables the control of the above-mentioned physicochemical properties of the catalysts [21]. 

Firstly, the metal precursor often determines the size of the particles and the metal–support interaction, these properties however often depend on the support and the preparation methods. Donphai et al. showed that the Ni catalyst prepared from acetate is characterized by smallest particle size on the silica surface compared to the systems where nitrate and carbonate were used as precursor. Moreover, using acetate and nitrate resulted in stronger interaction with the support surface than using carbonate [22]. This is in agreement with other works on Ni/SiO_2_ catalyst, where the use of Ni nitrate resulted in the formation of larger Ni crystallites in comparison to other precursors [23]. However, this stays in contrast to catalysts supported on different oxides. Ni introduced from nitrate had better dispersion and interacts more strongly with the alumina surface than when acetate was used [24]. On the other hand, for the same Ni/Al_2_O_3_ catalyst, Ni acetate enabled a higher dispersion and small Ni particle size compared to the counterparts derived from NiCl_2_, Ni(Acac)_2_ and Ni(NO_3_)_3_ precursors [25]. 

Despite the importance of the Ni precursor, this aspect has been marginally considered in the hydrogenation reactions of LA and HMF. In the case of HMF, to the best of our knowledge, there is no study concerning the effect of the Ni precursor. In the case of the hydrogenation reaction of LA to GVL, Lv et al. focused on various parameters influencing the Ni/MgO catalyst preparation conditions from nitrate precursor [26]. They evaluated both the effect of the reaction conditions, particularly the solvent effect, as well as that of different precipitation agents. The highest activity of Ni/MgO was attributed to the high metal dispersion that could be obtained thanks to the use of carbonate as a precipitation agent [26].

Herein, the influence of the Ni precursor on the activity in the hydrogenation reactions of biomass-derived LA and for HMF is presented. Nickel was deposited on aluminum oxide surface from both organic Ni(Acac)_2_, Ni(CH_3_COO)_2_, Ni(HCOO)_2_ salts, and inorganic NiCl_2_ and Ni(NO_3_)_2_ salts. It has been shown which factors of the preparation step influence the catalyst properties and to what extent it affects their performance in the hydrogenation of LA and HMF. The physicochemical characterization of the catalysts enabled assessing the key parameters driving the catalyst activity in both reactions. The structural properties of the Ni/Al_2_O_3_ catalysts are crucial in the LA hydrogenation reaction, while the nature of the interaction of nickel with the alumina surface affects the pathways of the HMF hydrogenation reaction.

## 2. Results

### 2.1. Thermal Decomposition of Catalysts Precursors

**Figure 1:**
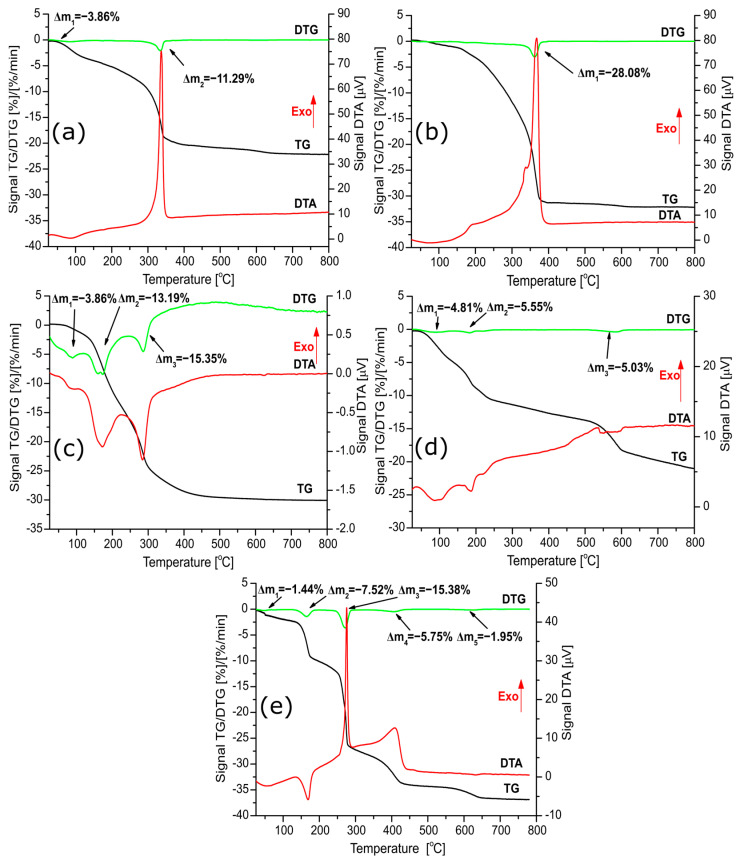
TG (black line), DTG (green line) and DTA(red line) curves for the catalyst precursors: (**a**) nickel acetate NiAc, (**b**) nickel acetylacetonate NiAcac, (**c**) nickel nitrate NiN, (**d**) nickel chloride NiCl, and (**e**) nickel formate NiF.

Figure 1 shows the thermogravimetric analysis of various nickel precursors deposited on the alumina surface. By comparing the obtained thermogravimetric (TG) curves, the first derivative (DTG) and the differential thermal analysis (DTA) curves, differences in the decomposition of individual precursors can be observed.

The decomposition of NiAc and NiAcac catalysts showed similar behavior (Figure 1a,b). Both DTA curves revealed one distinct exothermic peak finishing at 400 °C. For the NiAc catalysts, a slight endothermic peak at approximately 90 °C was observed, consistent with the loss of water from the support or the partial decomposition of the surface acetate groups of the nickel precursor. The second stage in the temperature range of 300–400 °C probably resulted from the volatilization of the organic fraction of the metal precursor, probably in the form of CO, CO_2_ and other related gases. At this stage, a largest weight loss of approximately 16% was observed. Moreover, at the temperature of 500 °C, a slight weight loss was evidenced, which may be related to the formation of the intermediate product—NiCO_3_ [27,28]. As in the case of NiAc, the decomposition of NiAcac on the alumina surface begins with release of adsorbed water (approximately 200 °C). The slow decomposition of NiAcac starts at approximately 300 °C. Above this temperature, the decomposition accelerates and ends at 400 °C with a final weight loss of 32%. During NiAcac decomposition, a significant amount of CO is released. Taking this into account, there is a possibility of carbonization of the metallic Ni. The key release of CO most likely takes place at temperatures above 300 °C. Therefore, it is possible that the resulting metallic nickel has undergone a carbonization process at the high temperature [29]. In the case of NiF (Figure 1e), the TG curve shows both endothermic and exothermic effects. The first endothermic effect at 180 °C is related to the dehydration of the sample. The weight loss of the sample was approximately 9%. The next two exothermic peaks occur at higher temperatures (290 °C and 410 °C). At the temperature of 290 °C, there was a 15% weight loss of the sample. In this temperature formic acid and carbon dioxide are released as the main gaseous products of decomposition of metal precursor to metallic nickel. In the next step, at a temperature above 400 °C, Ni is probably reoxidized. It has been shown that the decomposition of NiF in air at temperatures above 300 °C leads to the formation of a mixture of nickel oxide and metallic nickel [30,31]. The NiN (Figure 1c) decomposes in several steps with a total weight loss of approx. 30% at 500 °C. In contrast to organic precursors, in the case of the nitrate precursor, its DTA curve shows only endothermic peaks. The first weight loss (14%) in the range of 50–200 °C corresponds to the removal of physically adsorbed water from the sample, while the second weight loss (15%) in the range of 250–320 °C with maximum in 280 °C can be attributed to the thermal decomposition of Ni(OH)_2_ to NiO. The high decomposition temperature of the precursor may indicate the high affinity of the nickel nitrate to the support [32,33]. The differential thermal analysis curve of NiCl similar to NiN showed only endothermic effects (Figure 1d). The first two steps in the temperature range of 80–200 °C with a weight loss of approximately 9% are most likely related to a slow two-step water loss. The remaining three peaks occuring in the temperature range of 230–800 °C are associated with the complex processes of dehydrochlorination, dechlorination and partial oxidation [34,35]. The weight loss of the sample in this temperature range is approximately 10%.

### 2.2. Temperature-Programmed Reduction (TPR-H_2_) of Ni Catalysts

**Figure 2:**
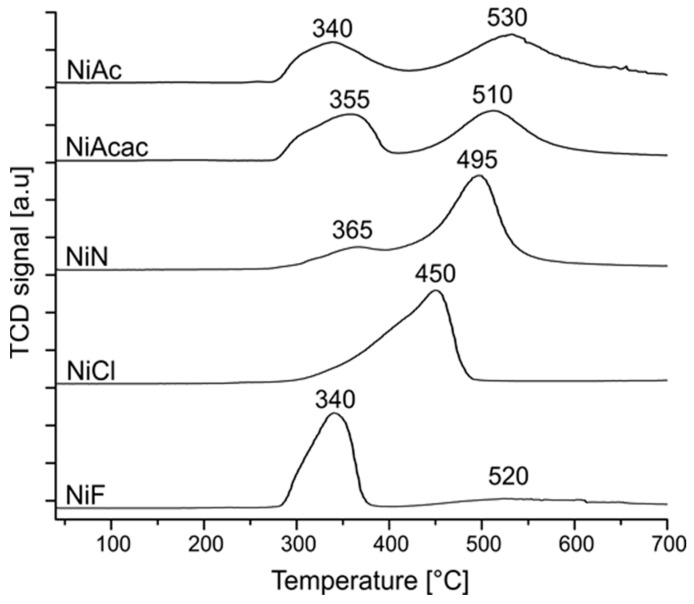
Temperature-programmed reduction profiles of Ni/γ-Al_2_O_3_ catalysts prepared from different Ni precursors.

The profiles of temperature-programmed hydrogen reduction of calcined nickel catalysts and the bare alumina support are presented in Figure 2. Character of the reduction peaks and their maximum temperature depend on the type of nickel precursor used during the catalyst preparation.

The profiles of NiAc and NiAcac catalysts have two clear effects of hydrogen consumption of similar intensity in the temperature ranges 250–400 °C and 400–650 °C. Hydrogen uptake in the range of lower temperature can be related to reduction of large nickel crystallites weakly interacting with the alumina surface while effect at higher temperature range can be assigned to the reduction of smaller nickel oxide particles strongly interacting with the support [36,37]. Similarly, to other catalysts with organic precursors the reduction of nickel species in the case of the NiF system takes place in two stages. The first major clear effect is visible in the temperature ranges 250–400 °C while second is much less intense in the range 400–700 °C. This suggests that larger metal crystallites weakly interacting with the support predominate on the surface of the NiF catalyst.

In contrast, only one dominant effect in high temperature is visible in the case of catalysts prepared with the use of inorganic precursors namely NiN (maximum at 500° with a shoulder at 350 °C) and NiCl (maximum at 440 °C) catalysts. This suggests that on the surface of both catalysts, there are small particles of nickel interacting with the support. The higher maximum temperature of NiN compared to NiCl indicates that the metal nanoparticles interact more strongly with the support on the catalyst surface prepared from the nitrate precursor. 

### 2.3. X-ray Diffraction (XRD) of Ni Catalysts

**Figure 3:**
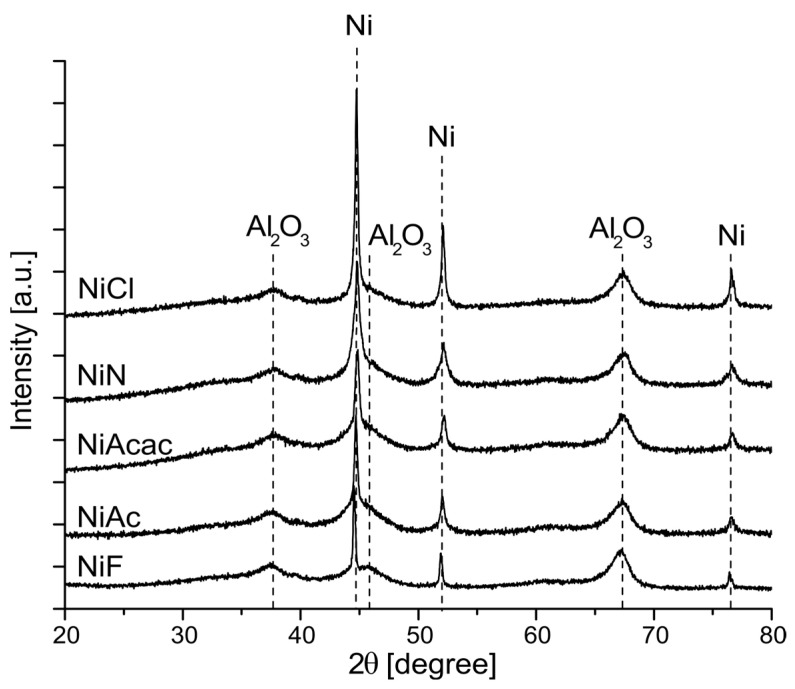
XRD patterns of Ni/γ-Al_2_O_3_ catalysts from different precursors reduced in 650 °C.

Figure 3 shows the diffractograms of the catalysts after reduction at 650 °C. The diffraction peaks at 37.5°, 45.7° and 66.6° correspond to the (311), (400) and (440) planes of the γ-Al_2_O_3_ polymorph (JCPDS 00–050-0741) [38]. Additional peaks at 44.5°, 51.8° and 76.4° are attributed to the planes (111), (200) and (220) of metallic nickel (JCPDS 04-0850) [39].

On the basis of the diffraction peaks assigned to nickel, the crystallite size of the metal was calculated using the Scherrer equation. The results are shown in Table 1. The NiF catalyst is characterized by the largest size of nickel crystallites (36 nm) on the alumina surface. On the other hand, the smallest nickel particle size (12 nm) was found for the NiN catalyst. The size of the nickel species on the surface of NiAc, NiAcac and NiCl catalysts was relatively similar, at 26, 20 and 26 nm, respectively.

### 2.4. Fourier Transform Infrared Spectroscopy (FTIR)

**Figure 4:**
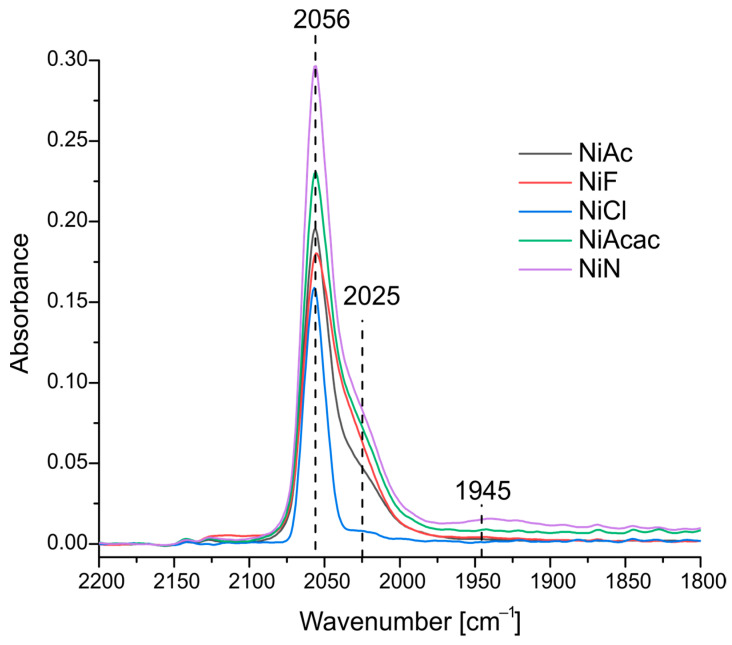
FTIR spectra of CO adsorbed on the Ni/γ-Al_2_O_3_ catalysts from different precursors reduced at 650 °C.

Carbon monoxide (CO) adsorption spectra (Figure 4) were recorded to assess the centers available on the surface of the nickel catalysts after reduction at 650 °C. Under the measurement conditions, no adsorption of CO was observed on a bare alumina in the wavenumber range of 2200–1800 cm^−^^1^. The spectra of all catalysts show a major band at 2056 cm*^−^*^1^ (L1), with a discernible shoulder at approximately 2025 cm*^−^*^1^ (L2). The L1 band is assigned to physically adsorbed CO on the nickel crystallites, while the L2 band is attributed to the linear chemisorption of CO on moderately dispersed metal crystallites [40,41,42]. Additionally, the spectrum of the NiN catalyst shows a band at 1945 cm*^−^*^1^ (B1) related to the bridge adsorption of CO [41,43]. The intensities of the bands indicate differences in terms of the quantity of the metallic centers on the surface of the catalysts. On the other hand, the intensity of the L1 band in the spectra of all catalysts decreases in the following order: NiN >> NiAcac > NiAc > NiF > NiCl. The intensity of the bands in the spectra of adsorbed CO correlates to the number of centers available on nickel species on the surface of the catalysts. The highest amount of metal centers is available on the surface of the catalyst prepared from inorganic nickel nitrate. In contrast, the catalyst from another inorganic nickel chloride precursor shows the lowest amount of metal centers on the catalyst. For catalysts obtained from organic precursors, the availability of nickel is similar to each other but slightly more metallic centers are present on the surface of NiAcac catalyst.

### 2.5. Temperature-Programmed Desorption of Ammonia (NH_3_-TPD)

The acidity of the catalysts and support was derived from the temperature-programmed desorption of NH_3_ and expressed as the µmolar amount of ammonia adsorbed per gram of sample (Table 2). The acidity of the Al_2_O_3_ is attributed to the presence of Al^3+^ Lewis acid sites in octahedral and tetrahedral symmetry [44]. The highest acidity was shown by the NiF catalyst (1090 µmol/g), and the lowest by the NiCl catalyst (670 µmol/g). The high acidity of the NiF may result from the formation of a strong interaction of nickel with the support or from the formation of a spinel which can lead to an increase in acidity due to the higher net positive charge or the coordination of unsaturated sites on the surface [45]. In the case of a NiCl catalyst, the presence of residual chlorine may affect the dispersion of the metal during calcination step, also residual chlorine can strongly adsorb on active sites and these factors may therefore contribute to the decrease in the acidity of the catalyst [46]. In other cases, the acidity was 865–980 µmol/g and was similar to the acidity of the bare alumina (980 µmol/g).

### 2.6. Catalytic Tests

The influence of the metal precursor on the activity of nickel catalysts was tested in the hydrogenation reactions of two promising biomass-derived chemicals, namely levulinic acid and hydromethylfurfural. The results of LA hydrogenation are presented in Table 3. The highest conversion of LA and yield of GVL was noticed for the catalyst NiN. The catalyst from nickel nitrate showed 76% of LA conversion and 73% of GVL yield. In contrast, the lowest activity was found for NiF and NiCl catalysts. In the case of both catalysts, the conversion of the substrate did not exceed 10%. In contrast to the NiF system, NiAc and NiAcac catalysts prepared from other organic precursors of the metal were more active in the reaction of LA hydrogenation. The NiAcac catalyst achieved 39% LA conversion and 30% yield of GVL. In turn, the catalyst obtained from NiAc showed 24% substrate conversion with 18% yield of GVL. As no other products were identified in the reaction mixture, the small difference between conversion and yield, observed in particular in the case of low activity catalysts, is most probably related to the adsorption of GVL on the surface. In agreement with DFT calculations, Huang et al. proved that adsorption of GVL is occurring mainly on the alumina support rather than on the Ni or Cu sites in the presence of a non-polar hydrocarbon-type solvent [47]. In polar solvents, this phenomena was also report to occurs, albeit to a lower extent This behavior was also reported for other supports and was shown to be significant at lower conversion [48].

The results of hydrogenation of hydroxymethylfurfural on nickel catalysts are presented in Table 4. Nearly total substrate conversion was achieved for the reaction with almost all catalysts. The exception is the NiCl catalyst which showed only 28% conversion of HMF. Nevertheless, the DMF (11% yield) was the main product in the presence of the NiCl catalyst. In the case of the catalyst from nickel formate, the same main reaction product was observed and 45% yield of DMF was noted. However, it is worth noting that the reaction with the NiF catalyst showed 22% of side products. In contrast to the NiCl system, the catalyst from nickel nitrate enabled 97% HMF conversion and 54% yield of BHMTHF. Additionally, in the presence of the NiN catalyst, 29% yield of MTHFA and small amount of DMTHF (8% yield) were obtained. In the case of catalysts obtained from the organic nickel precursors like NiAc and NiAcac, similar yields to the products of HMF hydrogenation were observed. The main products in both reactions were BHMTH and MTHFA. In addition to these two products, DMTHF was also noticed. Despite similar selectivity obtained on the NiN, NAcac and NiAc catalysts, differences in the yields to individual products are noticeable. Catalysts from organic precursors showed almost a 2-fold higher yield of DMTHFA (further hydrogenation product) compared to the NiN catalyst.

## 3. Discussion

The influence of the nickel precursor on the physicochemical and the catalytic properties of Ni/γ-Al_2_O_3_ catalysts was investigated in the hydrogenation reaction of two selected platform molecules, namely LA and HMF. Testing the catalysts in two reactions enabled a broader understanding of the relationship between the physicochemical properties and the activity of the obtained catalytic systems.

Both the type and the nature of the nickel precursors used in the catalyst preparation stage have an important impact on the physicochemical properties of catalysts, in particular on the size and the nature of metal crystallites. In the case of NiN and NiCl catalysts obtained from inorganic Ni precursors, a gradual decomposition of the metal precursor occurs during calcination accompanied by an endothermic effect, i.e., heat absorption. In contrast, when it comes to NiAc, NiF and NiAcac catalysts, the exothermic effects during the calcination of the organic precursor leads to local overheating at the surface and may result in an increase in the size of the nickel crystallites [49,50]. This suggests that the combustion of the residual organic precursor is responsible for the formation of nickel crystallites with larger sizes. As a consequence, the smallest metal crystallites on the support surface were found for the catalyst obtained from nitrate in comparison to organic precursors [51]. This phenomenon, a so-called shielding effect, is a result of the adsorption of metal ions in the vacant sites of the support, which leads to the formation of positive charge compensated by the anion of metal precursor. Anions lead to a limitation of available sites on the surface of the support for subsequent metal ions. The shielding effect of the anion is more pronounced for organic metal precursors due to its larger size [52].

**Figure 5:**
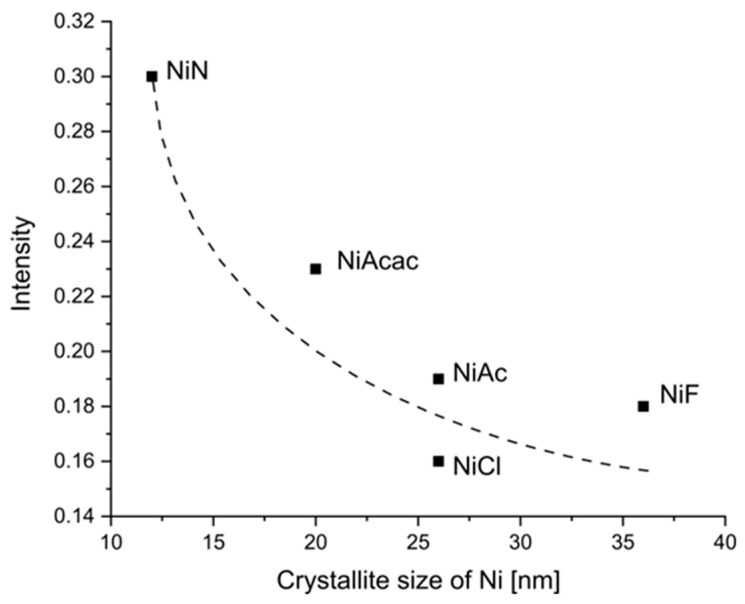
Influence of the L1 band intensity related to CO adsorption on Ni crystallites, on the crystallite size of this metal.

On the other hand, the largest size of the nickel crystallites was noticed on the surface of the NiF catalyst. The size of the metal crystallites determines the number of centers available on the catalyst surface as confirmed by FTIR spectra of CO adsorption. Figure 5 shows the relationship between the intensity of the main adsorption band (L1) in FTIR spectra and the size of the metal crystallites.

The largest number of centers is available on the surface of the NiN catalyst, while the smallest number of centers is on the surface of the NiF catalyst. It is worth noting that despite the similar sizes of nickel crystals on NiAc and NiCl catalysts, the latter is characterized by a lower availability of centers on the metal surface. This is probably due to the presence of residual chlorine, which strongly adsorbs on the active sites and poisons the catalyst [24]. It is also worth noting that the nickel precursor and consequently the size of the nickel crystallites affect the nature of their interaction with alumina. Large metal crystallites on the NiF catalyst interact poorly with the support surface. Reducing the size of the crystallites increases the interaction of the metal and alumina.

**Figure 6:**
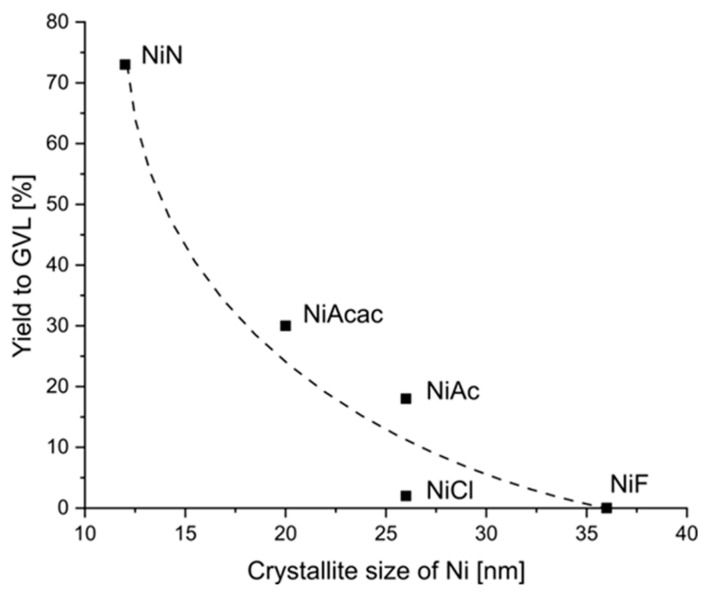
Influence of the Ni mean crystallite size on the yield of GVL for the Ni/γ-Al_2_O_3_ catalysts prepared from different precursors.

The physicochemical properties, in turn, determine the activity of alumina-supported Ni catalysts in the hydrogenation reactions of LA and HMF. Figure 6 shows the relationship between the GVL yield and the Ni size for the Ni/Al_2_O_3_ catalysts obtained from various precursors.

The highest yield of GVL was noticed for the NiN catalyst obtained from the nitrate precursor, which is characterized by the smallest size of metal crystallites (12 nm). The in-crease in the size of the metal crystallites on the support surface caused a decrease in the catalyst activity in the LA hydrogenation reaction.

However, the HMF hydrogenation reaction is much more sensitive and even slight changes in the surface properties of the catalysts affect the reaction yields. The main products of the HMF hydrogenation reaction for NiN, NiAcac and NiAc catalysts are the products with the reduced furan ring (BHMTHF) and further products of its hydrogenolysis (MTHFA and DMTHF). On the other hand, for the NiF catalyst, DMF is the main reaction product. A large Ni particle size favors the hydrogenation of the aldehyde group, while a small Ni particle size facilitates the hydrogenation of the furan ring [53]. This explains why high selectivity towards BHMTHF was observed for the NiN catalyst, as it is characterized by the smallest size of metal particles.

However, the selectivity to BHMTHF on nickel catalysts involves the hydrogenation of unsaturated bonds (C=O and C=C bonds), for which the hydrogenolysis of C-O bonds should be suppressed [16]. The acid centers present on the catalysts can thus contribute to the decrease in the selectivity to BHMTHF, and the acid sites on the NiN catalyst are responsible for the hydrogenolysis of BHMTHF to further products. In the case of NiAc and NiAcac that possess a bimodal nature of nickel crystallites, the contribution of small crystallites seems to be dominant in the reaction, as also in that case BHMTHF is identified as the main product. However, the lower selectivity to BHMTHF when the catalysts are prepared with the organic precursors is related not only to the presence of moderate acid sites which are of similar strength like in the case of NiN, but also to large Ni crystallites. The slower rate of hydrolysis on the NiN catalyst may also result from the high adsorption rate of high-molecular-weight compounds on the catalyst centers [54,55].

The HMF hydrogenation reaction on the NiF catalyst follows most probably the same selectivity path as for the other systems. However, the lower hydrogenation activity of the large metal particles and the high acidity of the NiF catalyst are responsible for dehydrat-ing BHMF and shifting the reaction yield towards DMF, and these factors can also contribute to the large fraction of side products.

## 4. Materials and Methods

### 4.1. Catalysts Preparation

Nickel catalysts were prepared by the wet impregnation method using γ-Al_2_O_3_ (Fluka, Buchs, Switzerland) as a support. Nickel catalysts were obtained from various nickel precursors: nickel acetate Ni(CH_3_COO)_2_ (98% puriss, Chemland, Stargard, Poland), nickel acetylacetonate Ni(acac)_2_ (≥98.0 puriss, Merck, Darmstadt, Germany), nickel nitrate Ni(NO_3_)_2_ x ×6H_2_O (99.9% puriss, Chempur, Piekary Śląskie, Poland), nickel chloride NiCl_2_ x ×6H_2_O (≥98.0 puriss, Chempur, Piekary Śląskie, Poland) and nickel formate Ni(HCOO)_2_ x ×2H_2_O (97% puriss, Alfa Aesar, Ward Hill, MA, USA), which are designated as a NiAc, NiAcac, NiN, NiCl and NiF, respectively. The Ni wt.% content in the Ni/γ-Al_2_O_3_ catalysts was 15% wt. All samples were calcined at 400 °C for 5 h under flow of air with a temperature ramp rate of 5 °C min^−1^, and further reduced under H_2_ flow for 1 h at 650 °C, directly before the reaction.

### 4.2. Catalysts Characterization

TG-DTA (thermal gravimetry-differential thermal analysis). Thermal analysis TGA-DTA-MS, which includes thermogravimetric (TG) and differential thermal analysis (DTA) investigations, has been performed using derivatograph SETSYS 16/18, Setaram (Caluire-et-Curie, France). The TGA-DTA spectra were recorded in the air flow (40 cm^3^/min) for the range of temperature 20–800 °C, with a heating rate of 5 °C/min. The mass sample varied between 5 and 20 mg and the samples were tested in the corundum crucible. 

Temperature-programmed reduction (TPR-H_2_) was performed on the AMI1 system from Altamira Instruments (Cumming, GA, USA) equipped with a thermal conductivity detector for examining the reducibility of the catalysts. In the experiments, mixtures of 5 vol.% H_2_ and 95 vol.% Ar or 2 vol.% O_2_ and 98 vol.% Ar were used at a space velocity of 3.1 × 10^−^^9^ g s^−^^1^ cm^−^^3^ and using a linear temperature ramp of 10 °C min^−^^1^.

X-ray diffraction (XRD) measurements were collected using a PANalytical X’Pert Pro MPD diffractometer (Malvern PANalytical, Malvern, UK). The X-ray source was a copper long fine focus X-ray diffraction tube operating at 40 kV and 30 mA. Data were collected in the 5–90° 2θ range with a 0.0167° step. Crystalline phases were identified by references to ICDD PDF-2 (version 2004) database. All calculations were performed with X’Pert High Score Plus computer program.

Fourier Transform Infrared (FTIR) spectra of the adsorbed CO were recorded on a Nicolett 6700 spectrometer (Thermo Scientific, Waltham, MA, USA) equipped with a liquid nitrogen-cooled MCT detector and a diffuse reflectance environmental chamber (Specac Ltd., Oprington, UK). The catalyst was placed in a sample holder, reduced in situ at 650 °C in flowing 5% H_2_/Ar for 1 h, and cooled to room temperature under Ar flow before recording the background spectrum. CO adsorption was carried out under a pressure of 5 bars of 5% CO/Ar after saturation of the sample surface for 30 min, while the spectra were collected at ambient pressure after release of the CO pressure. All spectra were recorded at a resolution of 4 cm^−^^1^, accumulating 64 scans.

Temperature-programmed desorption of NH_3_ was used to study the acidity of the catalysts. The NH_3_-TPD experiments were carried out in a quartz flow reactor. Before all experiments, the catalyst surface was cleaned with a He flow at 500 °C for 30 min. The temperature was further cooled down to 100 °C, and NH_3_ was adsorbed on the catalyst surface at 100 °C for 15 min. Before NH_3_-TPD experiment, physically adsorbed NH_3_ was removed from the catalyst surface by treating the sample with He carrier gas for 15 min and subsequently cooled down to room temperature. The NH_3_-TPD experiments were carried out from room temperature to 500 °C using a linear temperature ramp 25 °C min^−^^1^. 

ICP-AES (inductively coupled plasma atomic emission spectroscopy Thermo-Scientific, Waltham, MA, USA) was used to assess the nickel content on the support surface. In each case, ICP analysis showed 15% ± 0.3% nickel in the catalysts.

### 4.3. Catalytic Activity

Amounts of 1 g of LA and 0.1 g of catalyst, pH_2_ = 20 bar and 30 mL of water were combined in a stainless steel autoclave (Berghof, Eningen, Germany), equipped with a Teflon insert with a volume of 45 mL. The temperature of the reaction was maintained at 190 °C for 1 h. At the end of the reaction, the reactor was cooled down, the remaining pressure was released and the reaction mixture was centrifuged to separate the catalyst and liquid products. Liquid products were analyzed by high-performance liquid chromatograph (Agilent Technologies 1260 Infinity, Santa Clara, CA, USA) equipped with a refractive index detector and a Rezex ROA column, and 0.0025 mol/L H_2_SO_4_ was used as an eluent.

The activity test in HMF hydrogenation was performed in a 60 mL stainless steel batch autoclave (Premex, Lyss, Switzerland). The reaction was carried out with 1 g of HMF, 0.15 g of the catalyst and 30 mL of 1,4-dioxane under 30 bar of hydrogen. Then, the autoclave was heated to 220 °C and this temperature was maintained for 1 h. The reaction mixture was stirred at 650 rpm. Then, the reaction was cooled down to room temperature. The reaction mixture was centrifuged to separate the liquid sample and the catalyst. The reaction mixture was analyzed using a GC Agilent 7820 A (Santa Clara, CA, USA) equipped with a FID detector and high-polarity wax column Agilent CP-Wax 52 CB.

## 5. Conclusions

The effect of different metal precursors on the physicochemical and catalytic properties of Ni/Al_2_O_3_ was investigated. Thermal effects during the calcination of the catalysts determined metal dispersion, reducibility and metal–support interaction. The endothermic effects during the calcination of the inorganic precursor of nickel lead to a better dispersion and a stronger interaction of the metal with the surface of alumina. There is a clear relationship between the metal dispersion, the number of active sites, and the activity in the LA hydrogenation reaction. The catalyst with the smallest metal particles showed the highest activity in the LA hydrogenation reaction. On the other hand, both the size of crystallites and the acidity influence the path of the HMF hydrogenation. A large Ni particle size favors the hydrogenation of the aldehyde group, while a small Ni particle size and moderate acidity facilitate the hydrogenation of the furan ring, enabling reaching a high yield of BHMTHF.

## Figures and Tables

**Table 1:** Ni particle size value of the reduced catalysts at 650 °C calculated on the basis of XRD.

Catalyst	Ni Particle Size [nm]
NiAc	26
NiAcac	20
NiN	12
NiCl	26
NiF	36

**Table 2:** The acidity of Ni/γ-Al_2_O_3_ catalysts from various precursors investigated by NH_3_-TPD desorption.

Catalyst	Acidity [µmol/g]
NiAc	865
NiAcac	980
NiN	925
NiCl	670
NiF	1090

**Table 3:** Activity of Ni/γ-Al_2_O_3_ catalysts in LA hydrogenation with external hydrogen source.

Catalyst	LA Conversion [%]	GVL Yield [%]
NiAc	24	18
NiAcac	39	30
NiN	76	73
NiCl	9	2
NiF	8	0

Reaction conditions: 190 °C, 1 h, pH_2_ = 20 bar, 1 g of LA, 0.1 g of catalyst, 30 mL H_2_O; calcination temperature: 400 °C/5 h, reduction temperature: 650 °C/1 h.

**Table 4:** The activity of Ni/γ-Al_2_O_3_ catalysts in the HMF hydrogenation, expressed in terms of HMF conversion and different product yields.

Catalyst	Conversion [%]	Product Yield [%]							
	HMF 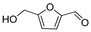	5-MF 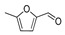	BHMF 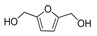	5-MFA 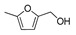	DMF 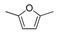	BHMTHF 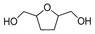	MTHFA 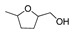	DMTHF 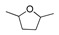	Others
NiAc	97	0	1	0	0	43	32	18	3
NiAcac	97	0	0	0	0	42	39	14	2
NiN	97	0	1	0	0	56	26	7	7
NiCl	28	8	5	4	11	0	0	0	0
NiF	100	0	8	11	45	14	0	0	22

Reaction conditions: 220 °C; 1 h; 0.3 g of catalyst; 1 g HMF, 30 mL 1,4-dioxane and 30 bar H_2_.

## Data Availability

The data will be available on request.
